# Impacts of three inspiratory muscle training programs on inspiratory muscles strength and endurance among intubated and mechanically ventilated patients with difficult weaning: a multicentre randomised controlled trial

**DOI:** 10.1186/s40560-024-00741-3

**Published:** 2024-07-25

**Authors:** Thomas Réginault, Roberto Martinez Alejos, Roxane Coueron, Jean-françois Burle, Alexandre Boyer, Eric Frison, Frédéric Vargas

**Affiliations:** 1 Department of Critical Care Medicine and Anesthesiology, Bordeaux University Hospital and School of Medicine, Bordeaux, France; 2 Montpellier University Training School of Physiotherapy, Montpellier, France; 3 Department of Critical Care Medicine, Lyon University Hospital and School of Medicine, Lyon, France; 4 Department of Clinical Research Methodology, Bordeaux University Hospital and School of Medicine, Bordeaux, France; 5 Medical Intensive Care Unit, Hôpital Pellegrin, Centre Universitaire de Bordeaux, Place Amélie Raba Léon, 33000 Bordeaux, France

**Keywords:** Mechanical ventilation, Weaning, Inspiratory muscle training, Physiotherapy

## Abstract

**Background:**

Inspiratory muscle training (IMT) is well-established as a safe option for combating inspiratory muscles weakness in the intensive care setting. It could improve inspiratory muscle strength and decrease weaning duration but a lack of knowledge on the optimal training regimen raise to inconsistent results. We made the hypothesis that an innovative mixed intensity program for both endurance and strength improvement could be more effective. We conducted a multicentre randomised controlled parallel trial comparing the impacts of three IMT protocols (low, high, and mixed intensity) on inspiratory muscle strength and endurance among difficult-to-wean patients.

**Methods:**

Ninety-two patients were randomly assigned to three groups with different training programs, where each performed an IMT program twice daily, 7 days per week, from inclusion until successful extubation or 30 days. The primary outcome was maximal inspiratory pressure (MIP) increase. Secondary outcomes included peak pressure (Ppk) increase as an endurance marker, mechanical ventilation (MV) duration, ICU length of stay, weaning success defined by a 2-day ventilator-free after extubation, reintubation rate and safety.

**Results:**

MIP increases were 10.8 ± 11.9 cmH_2_O, 4.5 ± 14.8 cmH_2_O, and 6.7 ± 14.5 cmH_2_O for the mixed intensity (MI), low intensity (LI), and high intensity (HI) groups, respectively. There was a non-statistically difference between the MI and LI groups (mean adjusted difference: 6.59, 97.5% CI [− 14.36; 1.18], *p* = 0.056); there was no difference between the MI and HI groups (mean adjusted difference: − 3.52, 97.5% CI [− 11.57; 4.53], *p* = 0.321). No significant differences in Ppk increase were observed among the three groups. Weaning success rate observed in MI, HI and LI group were 83.7% [95% CI 69.3; 93.2], 82.6% [95% CI 61.2; 95.0] and 73.9% [95% CI 51.6; 89.8], respectively. MV duration, ICU length of stay and reintubation rate had similar values. Over 629 IMT sessions, six adverse events including four spontaneously reversible bradycardia in LI group were possibly related to the study.

**Conclusions:**

Among difficult-to-wean patients receiving invasive MV, no statistically difference was observed in strength and endurance progression across three different IMT programs. IMT appears to be feasible in usual cares, but some serious adverse events such as bradycardia could motivate further research on the specific impact on cardiac system.

*Trial registration* Clinicaltrials.gov identifier: NCT02855619. Registered 28 September 2014

## Introduction

Mechanical ventilation (MV) through an endotracheal tube (ETT) is a crucial life-saving technique in intensive care units (ICUs). However, this common treatment is associated with serious complications and costs, often directly linked to the duration of ventilation [1–4].

MV weaning phase constitutes 40–50% of the total MV duration and 19 to 31% patients experience difficult or prolonged weaning, depending on the classification system [5, 6]. Difficult-to-wean patients exhibit increased ICU and hospital stays, morbidity, and mortality [7–9] and represent an increased burden regarding healthcare costs.

Diaphragm weakness, characterised by impaired muscle strength and endurance without neurological deficiency, is a major factor that causes delayed weaning from MV [10]. The prevalence of diaphragmatic weakness is high in intensive care, affecting more than 60% of patients upon admission [11], 63% at the initiation of weaning process [12] and up to 80% of patients with prolonged weaning process [13].

Inspiratory muscle training (IMT) is suggested as a non-pharmacological therapeutic option [14] to improve inspiratory muscle strength and probability of weaning success without any deleterious side effects, generally using a threshold loading device.

Despite growing research regarding IMT programs [15–21], the overarching evidence supporting the efficacy of IMT to shorten MV duration remains limited. This was highlighted by a recent randomized controlled trial led by Bisset et al. [22] failing to demonstrate a benefit regarding MV weaning time. Very heterogeneous modalities in IMT programs are applied in the literature, including endurance training programs (many repetitions and low resistance) [23], strength training programs (few repetitions and highest tolerable resistance) [24], and unclassifiable programs [15, 25]. There is still a lack of knowledge to target the appropriate load of training while various levels of improvement may be dependent on the applied protocol.

To assess the optimal IMT protocol, we combined several parameters. First, endurance is a major feature of a healthy diaphragm because of the need of a perpetual activity which explains the normal diaphragmatic composition (55% of type I fibers, 20% of type IIA fibers and 25% of type IIB fibers). Second, increased inspiratory muscles strength could be required because of specific increase in airways resistance due to the tracheal tube and post-extubation laryngeal resistance. Therefore, we hypothesized that an innovative mixed intensity (MI) IMT program targeting both endurance and strength improvement would present a greater benefit than usually used IMT programs. Thus, this study aims to compare three different modalities of IMT (low, high, and mixed intensity) on inspiratory muscle strength and endurance among difficult-to-wean patients.

## Methods

This multicentre prospective, superiority single-blind randomized, clinical trial in three parallel groups was conducted in two medical ICUs at Bordeaux University Hospital and Lyon University Hospital, France between October 2016 and January 2020.

### Characteristics of participants

Inclusion criteria were:

- patients older than 18 years
- receipt of at least 18 h of controlled MV
- first failure of a spontaneous breathing trial (SBT) or failed extubation. SBT failure was defined by the presence of at least one of the following outcome: (1) change in state of consciousness (anxiety, agitation, drowsiness), (2) impaired oxygenation (cyanosis, PaO_2_ < 50 mmHg or SpO_2_ < 90% with FiO_2_ > 50%), (3) dyspnea and signs of respiratory distress (drawing, use of accessory respiratory muscles, tachypnea > 35/min), (4) hemodynamic changes (tachycardia heart rhythm > 140 beat/min, (5) arterial hypertension > 180 mmHg or an increase of more than 20%, (6) arterial hypotension < 90 mmHg, presence of arrhythmias). Failed extubation was defined by the need of reintubation within 48 h after extubation.
- presence of weaning criteria as defined in the European Consensus Conference in 2007, including sedation reduction, spontaneous breathing cycles, PaO_2_/FiO_2_ ≥ 150, absence of inotropes or vasopressors at high doses or increasing doses (< 1 mg/h), oxyhaemoglobin saturation (SaO_2_) > 90% with FiO_2_ ≤ 50%, PEEP ≤ 8 cmH_2_O, temperature between 36 and 39 °C, and Glasgow Coma scale ≥ 8 [5].

Non-inclusion criteria were hemodynamic or respiratory instability, severe ventricular arrhythmias, poor short-term vital prognosis, cardiac arrest with a guarded neurological prognosis, proven neurodegenerative pathology, tracheotomy, current pregnancy, a curatorship measure, a do-not-resuscitate order, or a lack of coherence to follow verbal commands with a best motor response item of the Glasgow Coma scale lower than 6.

### Randomization

An unbalanced randomisation ratio favoured the innovative MI group (2:1:1), based on a randomised, computer-generated list with stratification according to study centre and permuted blocks of varying sizes (4 and 8). Given that a greater benefit was expected in participants benefiting from the MI strategy, and that we wished to collect as much data as possible on this new strategy, we decided to implement an unbalanced randomization scheme at design stage.

Randomisation was performed using a web-based centralised system after confirmation of participant eligibility. For reasons of staffing and feasibility, assessors were not independent and blinded for the allocation. Both patients and physiotherapists were not blinded to treatment allocation, but physicians responsible for the extubation decision were blinded to the IMT protocol.

The study protocol was approved by an independent ethics committee (Comité de Protection des Personnes Sud-Ouest et Outre-Mer III; DC 2016/03; NCT02855619). The study was performed in accordance with the CONSORT statement and the Declaration of Helsinki.

Adverse effects were notified by the investigator to the security and vigilance unit who determined the attributability of the event under study and the need for a declaration to the competent authorities.

### Sample size

A statistical analysis plan was developed and validated by the trial steering committee prior to the final database lock and analyses. Anticipated increases in MIP were projected to be 12 cmH_2_O in the MI group, 9.7 cmH_2_O in the HI group, and 9.9 cmH_2_O in the LI group, as previously reported [23, 24], with a common standard deviation of 2.5 cmH_2_O. An increase of 2 cmH_2_O in the MI group was considered a minimum threshold for additional gain compared with the other groups, representing a 20% increase in inspiratory force measurement. The study was thus designed to detect a difference equal to or greater than this threshold of 2 cmH_2_O between groups. As the MI group was compared with each of the two other groups for the primary objective, the two-sided type I error rate was set at 0.025 using Bonferroni correction. With 80% power and an unbalanced 2:1:1 ratio, 88 participants were required (46, 23, and 23 for MI, HI, and LI groups, respectively).

### Interventions

**Fig. 1:**
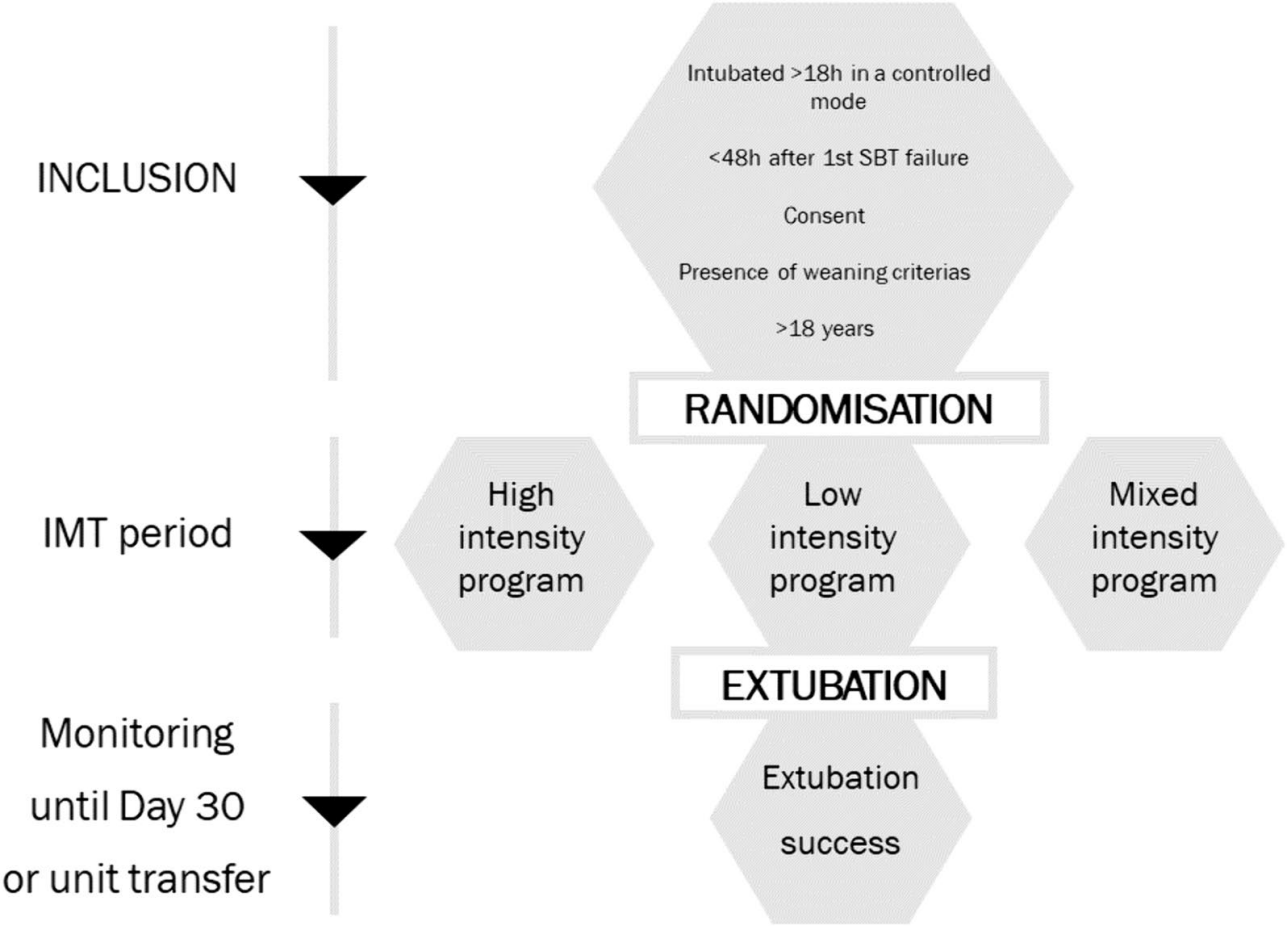
Study design. *SBT* spontaneous breathing trial, *IMT* inspiratory muscle training, *D* day

Study design is represented in Fig. 1.

**Fig. 2:**
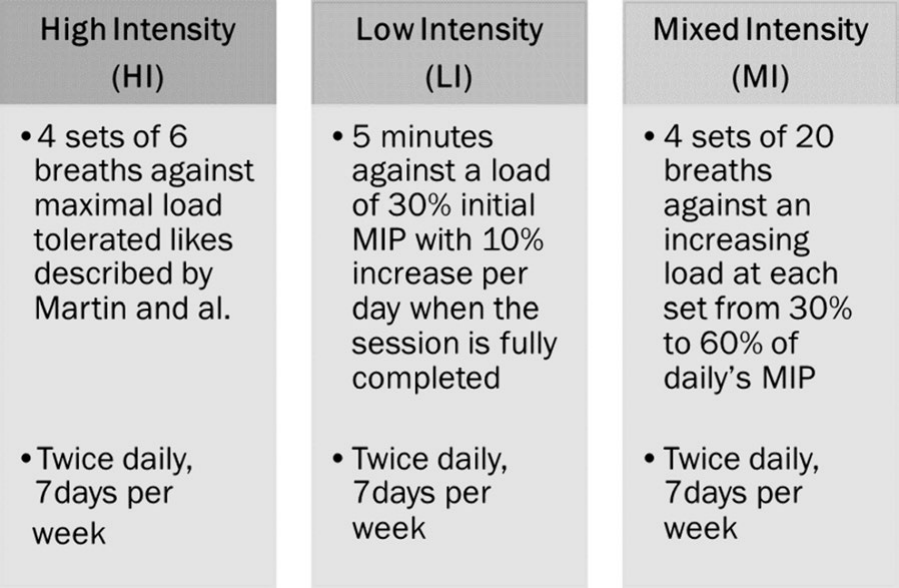
Description of the 3 IMT programs

Patients were enrolled within 48 h of the first failed SBT or failed extubation if they met the aforementioned eligibility criteria and consent had been obtained from the next of kin. Subsequently, they were randomised into one of the three IMT protocol groups (Fig. 2).

All participants received two IMT interventions daily (with ≥ 4 h between sessions) 7 days per week, from enrolment until one of two possible endpoints—successful extubation or 30 days of mechanical ventilation (D30), whereas arrived first. Considering potential fluctuations in consciousness or general stability, the inclusion criteria were assessed daily; patient participation in IMT sessions was contingent upon meeting these criteria. Before each training session, patients were positioned in a 45° Fowler’s position, and cardiorespiratory variables were evaluated to ensure hemodynamic stability. Criteria for instability included respiratory rate (RR) > 35 breath/min, SaO_2_ < 90%, systolic blood pressure (SBP) > 180 mmHg or < 90 mmHg, paradoxical breathing, agitation, and/or tachycardia.

Maximal inspiratory pressure (MIP; cmH_2_O) was measured daily before the first IMT session to obtain primary outcome data, although this measurement had the potential to induce a training effect and/or fatigue. All patients were disconnected from MV and performed IMT using the Threshold IMT device (Philips Respironics; Murrysville, PE, USA), directly connected to the ETT. Supplementary oxygen was added as needed. All patients were instructed to take large deep breaths from functional residual capacity and to breathe in with more force when it became difficult to open the IMT Threshold valve.

Between each set, or in case of instability as defined earlier, or if patient desire to discontinue the intervention, training sessions were halted and patients were reconnected to MV with previous pressure-support ventilation settings.

HI IMT program [24]: patients completed four sets of six breaths against the highest inspiratory resistance tolerated. Each series was interspersed with a 2-min pause during which patients were reconnected to MV. Resistance titration was initiated at 9 cmH_2_O (lowest load possible on the Threshold IMT device) on day 1. Subsequent adjustments of + 0, + 2, or + 5 cmH_2_O were made for each set according to patient tolerance and physiotherapist evaluation. Regardless of the daily MIP measurement, sessions began using the highest resistance from the previous day, with increases of + 0, + 2, or + 5 cmH_2_O for each set. Each set was interspersed with a 2-min pause to reconnect the patient to MV without parameter changes.

LI IMT program [23]: The physiotherapist applied a single inspiratory resistance equivalent to 30% of the MIP measured on the inclusion day for 5 min. The load was increased daily by 10% of the initial MIP, independent of the daily MIP measurement, if the previous session was completed. According to the American Sport College Medicine [26], for local muscular endurance training, it is recommended that light to moderate loads (40–60% of 1 maximal resistance) be performed for high repetitions (> 15) using short rest periods (< 90 s). To our point of view, this regimen of training performed by Cader et al. correspond to a low intensity program for endurance training.

MI IMT program: In this novel protocol, the physiotherapist set the inspiratory resistance device to 30% of the daily MIP for the first set of 20 breaths. In each set, resistance was increased by 10% of the daily MIP until a resistance equivalent to 60% of the MIP was achieved in the fourth set. Each set was interspersed with a 2-min pause to reconnect the patient to MV without parameter changes. This mixed regimen (both strength and endurance training have been inspired by the works of Bird et al. [27].

### Measurements

The primary outcome was the change in strength between the randomisation day (D1) and successful extubation or Day 30, using MIP (cmH_2_O) based on the method of Caruso et al. [28]. A unidirectional expiratory valve attached to the ETT and an external pneumotachograph (Fluxmed GrH monitor; MBMED, Buenos Aires, AR, USA) were used. MIP was daily assessed before the first session.

The main secondary outcome was endurance using an incremental endurance test to obtain peak (Ppk; cmH_2_O) [29]. For this test, patients breathed through an IMT Threshold device beginning at 30% of the initial MIP and increasing by 10% every 2 min until the effort was no longer tolerable. The maximum pressure tolerated for 2 min by the patient constituted the Ppk. This incremental endurance test was expressed with Ppk time corresponding to the test duration from beginning to task failure. The patient’s tolerance limit was the same used for instability during IMT as described above. This test, similar to a training stimulus, was only performed in each group on the first day of inclusion, then weekly and immediately before extubation. We think that this test could help for the assessment of inspiratory muscles even if, to our knowledge, this is the first time this test has been used on ICU population.

Other secondary outcomes included MV duration (number of days between inclusion and successful extubation, with no reintubation for > 48 h), ICU length of stay (days), weaning success defined by a 2-day ventilator-free after extubation, reintubation rate, and safety (occurrence of adverse events related to the study, numbers of completed IMT sessions).

### Statistical analysis

The primary analysis was conducted on an intent-to-treat basis, utilising the “last observation carried forward” (LOCF) strategy to replace missing data. In cases where MIP values were missing on Day 30 or successful extubation, the change in MIP between baseline and the last available follow-up value was used. If a participant experienced failed extubation on D30 with an end-of-study MIP measurement on the same day, the MIP value before extubation was used. Three patients who were tracheostomized before Day30 were considered in premature termination of monitoring and were treated using the LOCF strategy. The primary outcome was compared using a linear regression model adjusted for study centre (stratification variable), baseline MIP centred on the median, and both the presence of respiratory pathology at inclusion (defined as chronic obstructive pulmonary disease—COPD, asthma, chronic respiratory failure other than COPD, and neuromuscular disease), and length of MV before inclusion, which were regarded as a prognostic factor for extubation failure.

The same analytical strategy (including LOCF) was used to compare Ppk between Day 1 and successful extubation or Day 30. One participant with a missing baseline Ppk value was excluded from these analyses. Analysis of the other secondary outcomes was performed on participants with available data for variables included in the regression models, without imputation of missing data (i.e. complete cases analyses).

The primary analysis was performed using a two-sided overall type I error rate of 5%, with a *p* value threshold of 2.5% for each of the two comparisons (MI vs. LI and MI vs HI). A hierarchical testing procedure was predefined in the statistical analysis plan where for any particular comparison, if the null hypothesis for the primary endpoint (MIP) was rejected, then a statistical comparison of the secondary endpoint (Ppk) was performed at the same two-sided alpha threshold of 2.5%. If the null hypothesis for the primary outcome was not rejected, no hypothesis testing regarding Ppk was performed for that comparison. No hypothesis testing was conducted for the remaining secondary outcomes. All statistical analyses were performed using SAS v.9.4 software (SAS Institute).

## Results

### Participant flow

**Fig. 3:**
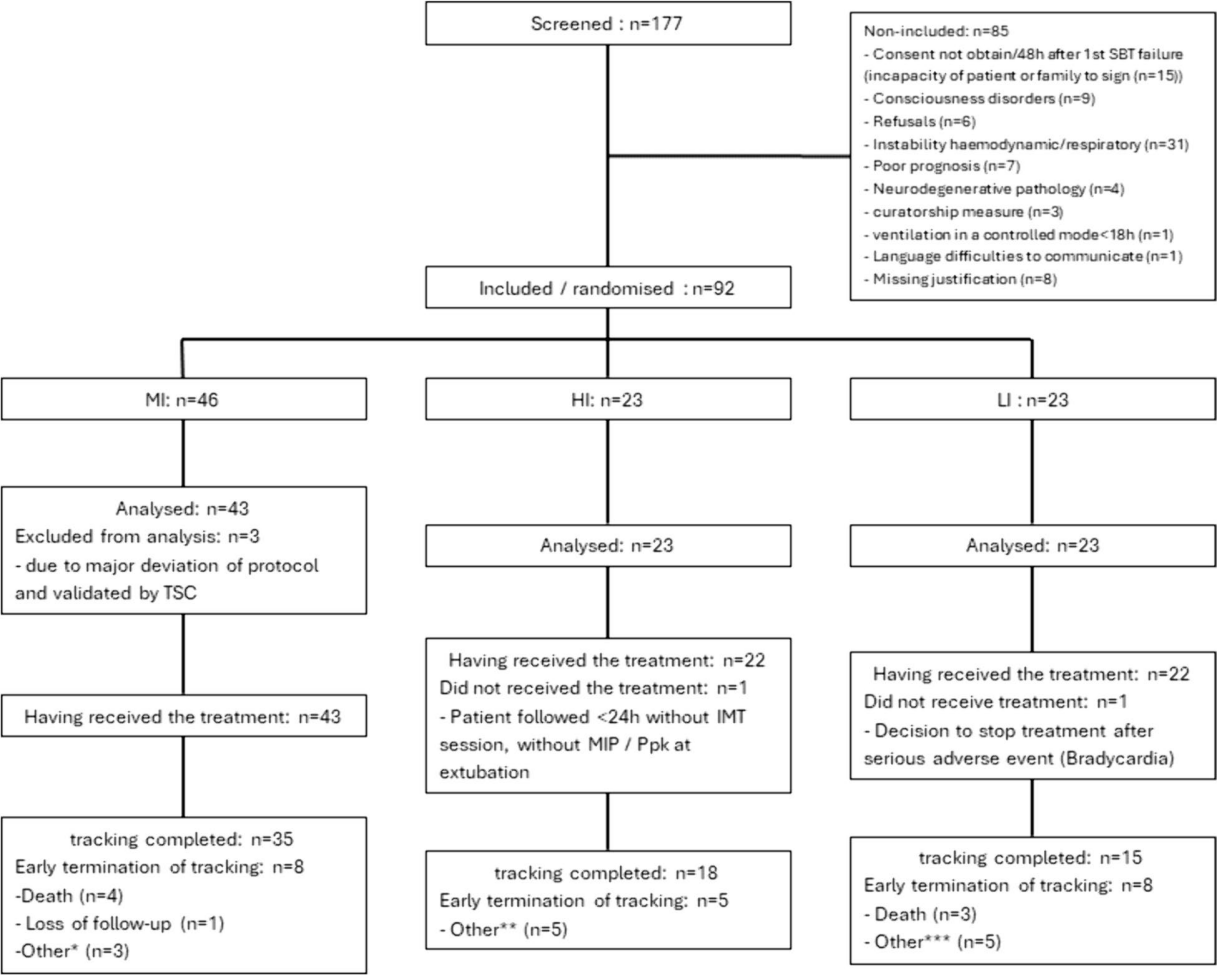
Flow chart of the study. *1 tracheostomy and 2 auto-extubations; **1 tracheostomy, 2 transfers, 1extubation < 24 h without any IMT sessions, and 1 pneumothorax unrelated to IMT; ***1 tracheostomy, 1 auto-extubation, 1 refusing MIP measurement, 1 severe bradycardia and 1 moderate isolated bradycardia; *SBT* spontaneous breathing trial, *MI* mixed intensity, *HI* high intensity, *LI* low intensity, *TSC* trial steering committee, *IMT* inspiratory muscle training, *MIP* maximal inspiratory pressure

In total, 177 patients with difficult weaning were screened between October 2016 and January 2020, and 92 were included in the study (Fig. 3). Three participants were excluded due to violation of major eligibility criteria, all in MI group. One patient had a degenerative pathology, one did not meet the blood gas inclusion criteria due to an erroneous result, and one exhibited hemodynamic instability immediately after inclusion. 89 participants were included in the final analyses. 21 patients did not complete the study until successful extubation, Day 30, or were not included in follow-up due to major protocol deviation. In total, 68 participants were followed up for the entire weaning period and completed the study.

The baseline characteristics of the study participants are presented in Table 1. There were no significant differences between groups. The included patients had a mean age of 66 years, and most were men (64%). The mean admission Simplified Acute Physiology Score (SAPS2) was 64.1, the mean PaO_2_/FiO_2_ at inclusion was 241.6, and the mean duration of MV at inclusion was 7.7 days. In all groups, the mean initial MIP was − 49.7 (SD 17.4) cmH_2_O, and the mean initial Ppk was 17.5 (SD 7.1) cmH_2_O with a mean initial Ppk time of 2.2 (SD 3.3) minutes.

**Table 1:** Baseline characteristics of participants

		MI group	HI group	LI group
Anthropometric data				
Patients (number)	*n*	43	23	23
Age (years)	Mean (SD)	65.2 (10.6)	67.6 (11.3)	66.4 (9.8)
Gender (number, %)	Male% Female %	27, 62.8% 16, 37.2%	15, 65.2% 8, 34.8%	15, 65.2% 8, 34.8%
Pre-existing medical pathology	*n*, %	35, 81.4%	18, 78.3%	18, 78.3%
Cause of MV introduction				
ARDS	*n*, %	12, 27.9%	6, 26.1%	12, 52.2%
Postoperative	*n*, %	5, 11.6%	3, 13.0%	1, 4.3%
Cardiac failure	*n*, %	5, 11.7%	5, 21.7%	6, 26.1%
Pneumonia	*n*, %	14, 32.6%	14, 60.9%	10, 43.5%
Sepsis	*n*, %	13, 30.2%	10, 43.5%	7, 30.4%
Coma	*n*, %	12, 27.9%	3, 13.0%	5, 21.7%
Associated treatments				
Curare	*n*, %	21, 48.8%	11, 47.8%	9, 39.1%
Endotracheal tube size (mm)	Mean (SD)	7.5 (0.35)^a^	7.5 (0.4)	7.5 (0.14)
Other data				
SAPS II score at admission	Mean (SD)	65.0 (19.8)^b^	65.3 (15.1)	61.4 (17.8)
GCS at inclusion	Mean (SD)	10.7 (0.8)	10.9 (0.3)	10.8 (0.4)
Length of MV before 1st SBT (days)	Mean (SD)	6.4 (7.4)	9.9 (11.8)	8.0 (5.8)
Last PaO_2_/FiO_2_ before inclusion	Mean (SD)	247.2 (74.9)	236.7 (69.1)	236.1 (64.4)
MIP at inclusion (cmH_2_O)	Mean (SD)	49.4 (16.1)	51.7 (18.2)	48.2 (19.4)
Ppk at inclusion (cmH_2_O)	Mean (SD)	16.9 (6.9)	18.5 (7)	18 (7.9)^c^
Ppk time at inclusion (min)	Mean (SD)	2 (3.2)	2.3 (2.9)	2.6 (4.1)^c^

Data are presented as mean (standard deviation; SD) or number and percentage (%)

*MI* mixed intensity, *HI* high intensity, *LI* low intensity, *ARDS* acute respiratory distress syndrome, *SAPS II* Simplified Acute Physiology Score, *GCS* Glasgow Coma scale, *MV* mechanical ventilation, *SBT* single breathe trial, *PaO*_*2*_ arterial pressure of O_2_, *FiO*_*2*_ inspired fraction of O_2_, *MIP* maximal inspiratory pressure, *Ppk* pressure peak

^a^*n* = 41, ^b^*n* = 42, ^c^*n* = 22

### IMT sessions: tolerance and safety

In total, 629 IMT sessions were conducted, with a mean session duration of 14.5 ± 5.2 min including 5 min of rest after training to monitor cardiorespiratory recovery. In each group, concerning fully performed sessions, protocols have been rigorously followed as described in the method over the number of repetitions, set or duration. For 42.2% of the follow-up days in the entire cohort, two IMT sessions were performed daily. In the study design, only one IMT session have to be conducted on D1, extubation day, and every 7 days of follow-up for Ppk assessment, already constituting a training session. Most of the time on D1 and extubation day, the time of inclusion or extubation prevented the completion of an IMT session. All results are presented in Table 2.

**Table 2:** General IMT sessions assessments

	MI group	HI group	LI group
Days without any session (*n*, %)	104 (32.5)	35 (26.1)	27 (25.7)
Days with 1 session (*n*, %)	80 (25)	44 (32.8)	33 (31.4)
Days with 2 sessions (*n*, %)	136 (42.5)	55 (41)	45 (42.9)
Sessions performed (*n*, %)	352 (55)	154 (57.5)	123 (58.6)
Duration (min) (mean; SD)	15.5 (5.4)	14.9 (3.9)	10.6 (3.8)
Number of session/patient (mean; SD)	8.2 (8.7)	6.7 (6.3)	5.3 (4.5)
Sessions cancelled (*n*, %)	288 (45)	114 (42.5)	87 (41.4)
Major reasons (*n*, %)			
Ppk measurement day	67	34	38
Insufficient level of consciousness	100	18	3
PaO_2_/FiO_2_ < 150	16	7	8
Cardiorespiratory instability	23	5	9
Refusal	11	8	7
Late schedule	22	8	7
Incomplete sessions/total sessions (*n*, %)	141 (40.1)	28 (18.2)	83 (67.5)
Session end time (*n*, %)			
Set 1	34 (27.9)	17 (60.7)	–
Set 2	30 (24.6)	4 (14.3)	–
Set 3	42 (34.4)	7 (25)	–
Set 4	16 (13.1)	0 (0)	–
Minute 1	–	–	16 (23.2)
Minute 2	–	–	20 (29)
Minute 3	–	–	23 (33.3)
Minute 4	–	–	12 (17.4)
Minute 5	–	–	3 (4.3)
Major reasons for stopping the session (*n*, %)			
Unable to open the valve	49 (23)	13 (27.7)	12 (13)
SaO_2_ < 90%	30 (14.1)	4 (8.5)	7 (7.6)
SBP > 180 mmHg	27 (12.7)	5 (10.6)	29 (31.5)
RR > 35 apm	72 (33.8)	18 (38.3)	41 (44.6)
Patient request (excessive perceived effort)	32 (15)	7 (14.9)	8 (8.7)
Resistances used (mean; SD)			
Set 1	15.3 (5.2)	19.5 (8.5)	17.2 (8.3)
Set 2	20.1 (7.4)	21.2 (8.1)	–
Set 3	24.9 (9)	22.8 (8.1)	–
Set 4	28.9 (9.5)	23.6 (7.9)	–
Safety			
Occurrence of adverse events^a^ (*n*)	1	0	5

*IMT* inspiratory muscle training, *MI* mixed intensity, *HI* high intensity, *LI* low intensity, *SD* standard deviation, *Ppk* pressure peak, *PaO*_*2*_ arterial pressure of O_2_, *FiO*_*2*_ inspired fraction of O_2_

^a^Described in the paragraph above titled “IMT sessions: tolerance and safety”

The mean training resistances used for IMT across all days ranged from 15.3 ± 5.2 cmH_2_O (1st set) to 28.9 ± 9.5 cmH_2_O (4th set) in the MI group, 19.5 ± 8.5 cmH_2_O (1st set) to 23.6 ± 7.9 cmH_2_O (4th set) in the HI group, and 17.2 ± 8.3 cmH_2_O in the LI group.

Six adverse events possibly related to the study were recorded. In the MI group, one patient developed a non-serious case of polypnea after MIP measurement and before the IMT session; in the LI group, five patients experienced adverse events (three serious cases of bradycardia including one during MIP measurement and two during IMT, one non-serious case of arterial hypertension, and one non-serious case of isolated bradycardia during IMT).

### Strength and endurance evaluation

Mean MIP, Ppk time, MV duration, ICU length of stay, weaning success and reintubation rate are presented in Table 3.

**Table 3:** Results of IMT in each group

	MI	HI	LI
^a^Change in MIP (cmH_2_O) mean (SD)	10.8 (11.9)	6.7 (14.5)	4.5 (14.8)
^a^Change in Ppk time (min) mean (SD)	2.5 (3)	1.7 (2.2)	2.2 (2.8)
^b^MV duration (days) *n* (%)	6.7 (5.8)	5.8 (5.1)	5.5 (4.7)
^b^ICU length of stay (days) *n* (%)	11.4 (8.3)	10.9 (8.2)	11.1 (8)
^b^Weaning success *n* (%)	36 (83.7)	19 (82.6)	17 (73.9)
^b^Reintubation *n* (%)	3 (7.5)	2 (9.5)	3 (13.6)

*IMT* inspiratory muscle training, *MIP* maximal inspiratory pressure, *MI* mixed intensity, *HI* high intensity, *LI* low intensity, *Ppk* pressure peak, *SD* standard deviation, *CI* confidence interval, *MV* mechanical ventilation, *ICU* intensive care unit

^a^Results with LOCF missing data management

^b^Description based on available data

The mean MIP at Day 1 in the total sample was 51.3 ± 17.2 cmH_2_O. The mean Ppk and Ppk time at Day 1 for the all sample were, respectively, 17.5 (7.1) cmH_2_O and 2.2 min.

**Fig. 4:**
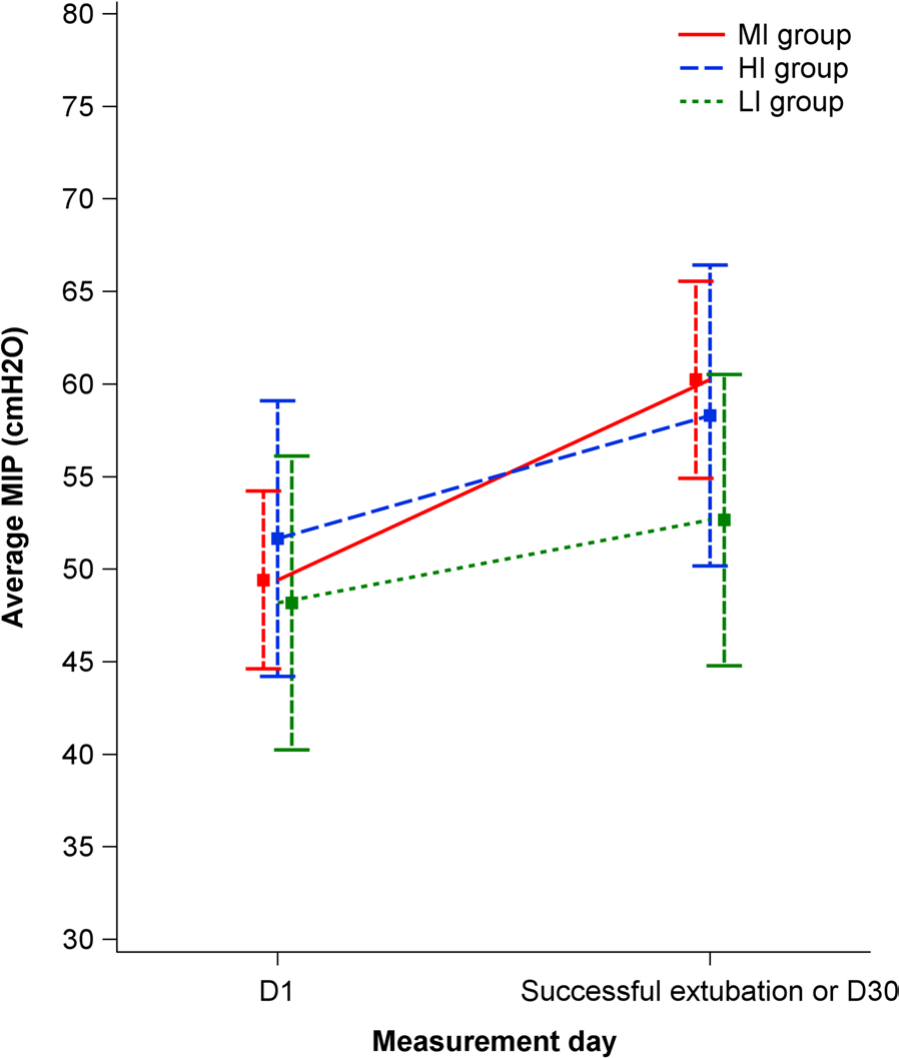
Mean MIP change in the IMT groups from baseline to D30 or successful intubation. *MIP* maximal inspiratory pressure; raw descriptive data at baseline and follow-up, with missing data imputed under last observation carried forward strategy and 97.5% confidence interval associated

Direct comparisons of MIP and Ppk changes between groups are presented in Table 4. Throughout the weaning process, there was a non-statistically significant difference in MIP change between the MI and LI groups (mean adjusted difference: − 6.59, 97.5% confidence interval (CI) [− 14.36; 1.18], *p* = 0.056); no difference was observed between the MI and HI groups (mean adjusted difference: − 3.52, 97.5% CI [− 11.57; 4.53], *p* = 0.321). The mean MIP change in each group is depicted in Fig. 4.

**Table 4:** Comparison of MIP and Ppk time changes between groups

	MI vs LI groups		MI vs HI groups	
	Mean adjusted difference [97.5% CI] *p* value*	Mean unadjusted difference [97.5% CI] *p* value*	Mean adjusted difference [97.5% CI] *p* value*	Mean unadjusted difference [97.5% CI] *p* value*
MIP	− 6.59 [− 14.36; 1.18] ***p* = 0.056**	− 6.61 [− 14.21; 1.00] ***p* = 0.051**	− 3.52 [− 11.57; 4.53] ***p* = 0.321**	− 3.67 [− 11.28; 3.94] ***p* = 0.274**
Ppk time	− 0.15 [− 1.72; 1.41]	− 0.17 [− 1.79; 0.84]	− 0.82 [− 2.41; 0.77]	− 0.75 [− 2.34; 0.84]

*MIP* maximal inspiratory pressure, *MI* mixed intensity, *HI* high intensity, *LI* low intensity, *Ppk* pressure peak, *CI* confidence interval

*p value are provided only for between-group comparisons on the primary outcome (MIP), as per the hierarchical testing procedure implemented to control for the overall type 1 error rate

Throughout the weaning process, the mean adjusted difference in Ppk change between the MI and HI groups was − 0.82 (97.5% CI [− 2.41; 0.77]), whereas the mean adjusted difference between the MI and LI groups was − 0.15 (97.5% CI [− 1.72; 1.41]).

## Discussion

To our knowledge, this study is the first to directly compare the impact of three different IMT modalities (low, high, and mixed intensity) on inspiratory muscle strength and endurance among difficult-to-wean patients, using MIP as a clinically relevant outcome. Our findings indicate that inspiratory muscles strength and endurance are increased during the weaning process for difficult-to-wean patients, regardless of IMT protocol used. We did not identify any statistically significant differences between the groups.

The non-significant increase in MIP in the MI groups must be related to the lack of strict selection approach for difficult-to-wean patients in this study, which might have led to heterogeneity within the sample. In our results, mean MIP at inclusion was 49.7 ± 17.4 cmH_2_O which is not consistent with a strong impairment of inspiratory muscles strength. It is difficult to determine the principal cause of SBT failure, which involves multifactorial processes with distinct interacting clinical conditions [30]. The introduction of a MIP cutoff as an inclusion criterion should have been performed to better target the difficult-to-wean population with inspiratory muscles weakness. A potential solution may be to use a MIP cutoff point, as described by De Jung et al*.* This study included 116 difficult-to-wean patients, whose characteristics were very similar to our populations, in particular they have received at least 7 days of MV. This study presented a higher rate of extubation failure when MIP was below − 30 cmH_2_O [31]. Another approach to choose the MIP cutoff value is to use the results of Tzanis et al*.* who have determinated a MIP cutoff value of − 36 cmH_2_O, below which weaning period is longer [32]. Consequently, it appears relevant to use one of these two MIP values in order to refine the target population for carrying out an IMT. Moreover, the observed heterogeneity in MIP progression was higher than anticipated during the study design process, which may have led to an underpowered study with non-statistically significant results, although the point estimates of MIP differences in favour of the MI group exceeded the predefined clinically meaningful threshold of 2 cmH_2_O.

We considered the IMT protocols presented by Cader et al. and Martin et al. to be effective and well-tolerated; however, we treated these protocols as active control groups, presuming that inspiratory muscles performances could be significantly improved with our novel IMT, which aims to increase both inspiratory muscles strength and endurance. In our replication of the protocol presented by Cader et al*.*, we observed a MIP increase of 5.3 ± 15.5 cmH_2_O, compared with 7.6 cmH_2_O (95% CI [5.8 to 9.4]) in the original study [23]. Similarly, in our reproduction of the protocol presented by Martin et al., we found an MIP increase of 6.8 ± 15.1 cmH_2_O, compared with 9.7 cmH_2_O in the original study [24]. These differences can be explained by the distinct populations studied. Cader et al. included older intubated patients (aged > 70 years) with very low initial MIP (< − 20 cmH_2_O), suggesting a larger MIP increase in patients with low baseline values. Martin et al. included difficult-to-wean tracheotomised patients who had received MV for a mean of 41.9 days. The timing of IMT initiation remains controversial. A recent systematic review and meta-analysis indicated that IMT in the ICU was initiated during early MV in eight studies, after proven difficulty in weaning and frequently in tracheotomised patients in 14 studies, and after extubation in three studies [18]. Bisset et al. provided a practical guide for performing IMT in the ICU, which suggests initiation after 7 days of MV [20]. In the present study, IMT was initiated immediately after the first SBT failure, selecting patients who required assistance to wean off MV; this approach corresponded to a mean of 7.7 days of MV, consistent with the recommendations of Bisset et al. Another explanation that the present study did not fully corroborate these findings, can possibly due to deviations performing Martin et al. and Cader et al. protocols in our study compared to the original reports to facilitate comparisons. Firstly, we used the Threshold IMT device (Philips Respironics; Murrysville, PE, USA) in all three groups while Martin and al. have used a threshold PEP (Philips Respironics; Murrysville, PE, USA) in order to start the first session at Day1 with a resistance equal to 5 cmH_2_O compared to the use of threshold IMT (Philips Respironics) forcing us to start the first session at Day1 with a resistance equal to 9 cmH_2_O. Secondly, IMT was performed twice a day if possible while Cader et al. performed IMT one per day in their original report.

In the present study, we aimed to make a consistent comparison between the three IMT protocols. We have reported many data on the progress of the sessions. Mean duration of session allows IMT to be easily included in usual cares. Mean duration of sessions, including 5 min of rest and monitoring, was similar between MI ant HI group with, respectively, 14.7 ± 5.4 min and 14.9 ± 3.9 min, but was lower in LI group with 10.6 ± 3.8 min. These results can be explained by 2-min rest periods between each set in both MI and HI group which extend the duration of sessions compared to the duration set at five minutes in LI group. It is also necessary to analyse the tolerance of patients to each protocol. Firstly, we focus on the number of incomplete sessions which is very high in the LI group with 66.9% versus 40.1% in MI group and 18.2% in HI group. HI group seems to be very well tolerated especially compared to the LI group. Secondly, we look at when and why the sessions stop. On average, we observed a majority of sessions end time after the third set correspond to a threshold of 50% of daily MIP in MI group, after the first set corresponding to the last higher threshold used in HI group, and after 3 min with a threshold equal to 30% initial MIP with an increase of10% when last session fully completed. The design of HI protocol with an increase to the maximal threshold tolerated can lead to intolerance what could be explained by it does not take into consideration the variability of patients' fitness level compared with MI protocol which use the daily MIP measurement to calibrate the threshold. This hypothesis is consistent regarding the reason for stopping the session which is 27.7% in relation with incapacity to open the valve in HI group, compared to 23% in MI group and 13% in LI group. Finally, six serious adverse effects have been reported with a majority of bradycardia, including 5 in the LI group. This unexpected finding could motivate further research on the specific impact of IMT on cardiac system.

This study had some important limitations. First, the absence of group control in this study constitute the main limitation and it was assumed that the IMT protocols presented by Cader et al. and Martin et al. were effective, well-tolerated and were treated as active control groups while the mixed program was assumed to have the potential for significant improvement of inspiratory muscles performance. It might be useful to compare these interventions with a standard of care that excludes any IMT intervention, which would better illustrate their relative efficacies. In the literature, a recent RCT performed by Khodabandeloo et al. [17] in a similar population have shown a mean increase in MIP of 13.7 cmH2O (95% CI [11.76–15.63] in the IMT group vs 9.08 cmH2O (95% CI [7.14–11.02] in the control group. This result corroborates those demonstrated by Vorona in a meta-analysis with a better recovery of MIP associated to IMT protocols even if a spontaneous recovery occurs without specific training [18]. At any rate, we acknowledge that a randomised controlled trial comparing IMT protocols to a true control group would be the appropriate way to confirm the added value of these protocols. Moreover, the lack of blinding also introduced the possibility of measurement bias.

Second, the expected improvement in MIP of 2 cmH_2_O in the innovative mixed intensity program, representing a 20% increase in inspiratory force measurement compared to the results obtained by Cader and Martin, remains a limitation due to the questioning of clinical relevance.

Finally, we have neglected assessment of quality of life as it often happens in clinical trials involving these patient populations. Future studies should explore the effects of IMT on short-term and long-term quality of life as a primary outcome. In addition, assessments of IMT impacts on hospital or ICU readmission rates may improve the understanding of its effects after the initial hospital stay.

Making a balance between efficiency, feasibility and tolerance of our results, even if the results indicated a trend favouring the mixed intensity program for both inspiratory muscle strength and endurance improvement, the choice of IMT protocol remains undecided and further research are needed.

## Conclusions

Among difficult-to-wean patients receiving invasive MV, no statistically significant differences were observed in strength and endurance progression across three IMT programs. IMT appears to be feasible in usual cares, well tolerated and safe, but few serious adverse events of bradycardia could motivate further research on the specific impact of IMT on cardiac system.

## Data Availability

Thomas Réginault and Frédéric Vargas had full access to all the data in the study and take responsibility for the integrity of the data and the accuracy of the data analysis.

## Abbreviations

ARDS: Acute Respiratory Distress Syndrome
EPAP: Expiratory positive airway pressure
ETT: Endo tracheal tube
FiO2: Inspired fraction of O_2_
GLS: Glasgow Coma scale
HI: High intensity
ICUs: Intensive Care Units
IMT: Inspiratory muscle training
MIP: Maximal inspiratory pressure
LI: Low intensity
LOCF: Last observation carried forward
MI: Mixed intensity
MV: Mechanical ventilation
PaO_2_: Arterial pressure of O_2_
Ppk: Peak pressure
RR: Respiratory rate
SaO_2_: Pulsed oxygen saturation
SAPS II: Simplified Acute Physiology Score
SBP: Systolic blood pressure
SBT: Spontaneous breathing trial
